# High rate of incidental detection of skin cancer following teledermatology consultation: Full body skin examination may detect more skin cancers than a referral-based approach

**DOI:** 10.1016/j.jdin.2023.08.013

**Published:** 2023-08-28

**Authors:** Trilokraj Tejasvi, Nicole Trupiano, Thomas Scharnitz, Taylor Novice, Charles N. Ellis

**Affiliations:** a Dermatology Service, Veterans Affairs Ann Arbor Healthcare System, Ann Arbor, Michigan; b Department of Dermatology, University of Michigan Medical School, Ann Arbor, Michigan; c Department of Dermatology, Henry Ford Health System, Detroit, Michigan

**Keywords:** incidental findings, melanoma, skin cancer detection, teledermatology, veterans

*To the Editor:* Store-and-forward teledermatology^1^ is widely used throughout the Veterans Affairs Health System for assessing high-risk skin lesions. In this method, skin lesions identified by primary-care providers are photographed and later evaluated by dermatologists at the Veteran’s Affairs Healthcare System, who may be located at substantial distance. Others have noted that both teledermatology^2^ and in-person^3^, ^4^, ^5^ examinations by consulting dermatologists detected skin cancers that had not been mentioned by referring providers. We assessed our detection of such cancers during in-person visits related to teledermatology consults.

In a 6-month retrospective quality assurance study at the Veterans Affairs Ann Arbor Health System, we reviewed all teledermatology consults, choosing those of patients who had biopsy of at least 1 non-rash lesion during subsequent in-person dermatology evaluations.

Among 181 patients (98% male, 85% White, average age 67 years) who met inclusion criteria, there were 175 biopsied lesions that had been photographed as part of the teledermatology consultation, and 132 biopsied lesions that were *incidental* (ie, had not been requested to be photographed and were discovered at in-person visits). Thus, on average, for every 10 biopsied lesions identified in teledermatology consults, we found 8 additional clinically-concerning incidental lesions during in-person evaluation. Among biopsied lesions, malignancy occurred in 55% (96/175) of those photographed and 61% (81/132) of those that were incidental. Therefore, in-person examinations yield 84% (81/96) more skin cancers than identified in teledermatology consultations among veterans.

As expected, rate of malignancies was greater on UV-exposed skin (we defined as head, neck, and distal to and including knees or elbows; 66% of these biopsies were malignant) compared to covered skin (47% malignant). Although we do not know which photographed lesions were identified de novo by patients’ providers or were pointed out by patients, lesions of exposed skin are more likely noticed by providers, particularly in many medical visits in which clothing is not removed, and by patients and their friends or family. Therefore, skin cancers under clothing will tend to be discovered incidentally during skin examinations.

For example, of 8 melanomas discovered, while 2 were photographed, 6 were incidental, yielding an incidental melanoma detection rate of 5% (6/132 biopsied lesions). The 6 incidental melanomas (75% of melanomas identified) occurred on back, chest, or abdomen.

Our study highlights risk of undetected skin malignancies, including melanomas, among veterans who have skin lesions that generate teledermatology evaluations and subsequent biopsies. Identifying more malignancies requires examinations of skin beyond that of initial lesions of interest to patients or primary-care providers.

Whether such evaluations could be provided effectively by primary-care providers or require in-person assessment by dermatologists is beyond the scope of our work; however, some otherwise unphotographed lesions might be identified for teledermatology assessments during expanded primary-care evaluations of skin. Indeed, we found 41 incidental malignancies on *exposed* skin, indicating that requesting providers did not recognize certain skin cancers or did not examine broadly enough.^2^^,^^5^

We also found 39 incidental malignancies on covered skin. High-yield areas for detecting incidental skin cancers, including melanomas, are back, chest, and abdomen particularly in men, suggesting the importance of at least a waist-up examination by referring physicians, waist-up photography in teledermatology referrals, or both.

## Conflicts of interest

Drs Tejasvi and Ellis are employees of the Department of Veterans Affairs. Author Dr Trupiano, Dr Scharnitz, and Dr Novice have no conflicts of interest to declare.
